# Proteomic Profiling of *Emiliania huxleyi* Using a Three-Dimensional Separation Method Combined with Tandem Mass Spectrometry

**DOI:** 10.3390/molecules25133028

**Published:** 2020-07-02

**Authors:** Goyeun Yun, Jong-Moon Park, Van-An Duong, Jeong-Hun Mok, Jongho Jeon, Onyou Nam, Joonwon Lee, EonSeon Jin, Hookeun Lee

**Affiliations:** 1 College of Pharmacy, Gachon University, Incheon 21936, Korea; ggo1203@hotmail.com (G.Y.); bio4647@naver.com (J.-M.P.); anduong@gachon.ac.kr (V.-A.D.); jeonghunmok@naver.com (J.-H.M.); jeonjh8817@naver.com (J.J.); 2 Basilbiotech, Seoul 06621, Korea; 3 Department of Life Science, Hanyang University, Seoul 04763, Korea; namonew@naver.com; 4 College of Letters and Science, University of California Los Angeles, Los Angeles, CA 90095, USA; joonwonlee7@gmail.com

**Keywords:** *Emiliania huxleyi*, three-dimensional (3D-LC) separation, proteomic profiling, gene ontology, photosynthesis

## Abstract

*Emiliania huxleyi* is one of the most abundant marine planktons, and it has a crucial feature in the carbon cycle. However, proteomic analyses of *Emiliania huxleyi* have not been done extensively. In this study, a three-dimensional liquid chromatography (3D-LC) system consisting of strong cation exchange, high- and low-pH reversed-phase liquid chromatography was established for in-depth proteomic profiling of *Emiliania huxleyi*. From tryptic proteome digest, 70 fractions were generated and analyzed using liquid chromatography-tandem mass spectrometry. In total, more than 84,000 unique peptides and 10,000 proteins groups were identified with a false discovery rate of ≤0.01. The physicochemical properties of the identified peptides were evaluated. Using ClueGO, approximately 700 gene ontology terms and 15 pathways were defined from the identified protein groups with *p*-value ≤0.05, covering a wide range of biological processes, cellular components, and molecular functions. Many biological processes associated with CO_2_ fixation, photosynthesis, biosynthesis, and metabolic process were identified. Various molecular functions relating to protein binding and enzyme activities were also found. The 3D-LC strategy is a powerful approach for comparative proteomic studies on *Emiliania huxleyi* to reveal changes in its protein level and related mechanism.

## 1. Introduction

*Emiliania huxleyi* (*E. huxleyi*), a member of the coccolithophores group (calcareous alga), is a unicellular marine phytoplankton that can be found throughout the ocean with a unique morphology [1]. It has been known to play a critical role in global biogeochemistry through the uptake of CO_2_ by photosynthesis and calcification [2]. Calcification is a biogenic process of calcium carbonate production in *E. huxleyi* and other coccolithophores. During calcification, *E. huxleyi* produces mineral plates consisting of calcium carbonate on exoskeletons known as coccoliths [3]. The sedimentation of these compartments in the ocean contributes negatively to ocean acidification [4]. *E. huxleyi* is able to form massive blooms covering the ocean surface up to 250,000 km^2^, which are sometimes detected by satellite. It reflects the amount of CO_2_ absorbed by the ocean and is closely related to climate changes on Earth [5]. As the biogeochemical and ecological importance of *E. huxleyi* has been recognized, various physiological, biochemical, and genetic studies have been extensively carried out with this species [6,7,8,9,10]. The genome sequence database of *E. huxleyi* (strain CCMP 1516) was previously constructed in 2013, which consisted of 30,569 protein-coding genes [11]. Nevertheless, proteomic analysis of this species has just been reported in a few studies with a small number of identified proteins. Jones et al. identified 99 proteins from *E. huxleyi* (strain NZEH) using one-dimensional SDS-PAGE and liquid chromatography-tandem mass spectrometry (LC–MS/MS) [12]. This group later utilized two-dimensional liquid chromatography (2D-LC) and identified 115 homologous protein groups from the same strain [13]. Another group used LC-MS/MS to identify 346 to 500 proteins from *E. huxleyi* (strain CCMP 1516) [14,15]. Thus, it is necessary to perform a proteomic profiling study on *E. huxleyi* to identify a large dataset of its protein identification.

Proteomics is the study of the entire proteins (proteome) in a sample. The analysis of highly complex samples has been a main technical task in proteomic research aiming at genome-wide analysis with the identification of low-abundance proteins [16]. Various approaches have attempted to achieve complete proteome coverage of complex samples, and yet reducing sample complexity remains a bottleneck against reaching a fundamental goal in proteomics [17]. In proteomic studies, separations in protein or peptide levels are frequently used to reduce sample complexity prior to mass spectrometric analysis [18,19,20,21]. Many separation techniques have been widely used in proteomic studies, including two-dimensional electrophoresis (2-DE) [22], reversed-phase liquid chromatography (RPLC) [23], isoelectric focusing [24], and capillary zone electrophoresis (CZE) [25]. To improve the identification of peptides and proteins, several multi-dimensional separation methods have been developed and evaluated [26,27,28]. Multi-dimensional separation is currently considered as one of the most powerful approaches to increase peak capacity [29,30,31]. The concept of multi-dimensional separation was described by Giddings in 1984, which stated that two or more independent separation methods could be coupled based on the orthogonality in elution mechanisms to resolve complex mixtures [32]. Based on that, the first Multi-dimensional Protein Identification Technology, an online proteomic technique packing strong cation exchange (SCX) and RP resins into a single capillary, was successfully developed [23,33]. Recently, an integrated spintip consisting of SCX beads and RP disk in one pipet tip was developed for deep proteomic profiling [34]. Multi-dimensional separation is classified into three main strategies [35,36]. The classic approach starts with the separation of proteins by 2-DE or LC prior to enzymatic digestion and LC-MS/MS analysis of the peptide digest [37]. The second strategy is applied in top-down proteomics, which allows the multi-dimensional separation in protein level, followed by MS/MS analysis [38]. The third method is widely used in bottom-up proteomics studies, which first digests the proteins into peptides prior to multi-dimensional separation and MS/MS analysis [39]. In bottom-up proteomics, SCX-RPLC has been widely used due to their high orthogonality, resulting in better separation and resolving power [40,41,42]. Besides, the combination of two RPLC under extremely different pH conditions showed the highest peak capacity among various chromatographic combinations [43,44]. The effectiveness of two-dimensional (2D) separation with high-pH (HpH) and low-pH (LpH) RPLC was confirmed through the increases in peptide and protein identification [45,46]. Some three-dimensional LC (3D-LC) platforms have been demonstrated. Wei et al. developed a 3D (RPLC-SCX-RPLC) system to analyze proteins in yeast and identified 5954 unique peptides and 1457 proteins [47]. Similar strategies were used for proteomic profiling of monkey brain tissue [48], human hepatocellular carcinoma tissues [49], and plasma of patients with sepsis and systemic inflammatory response syndrome [50]. Betancourt et al. combined SCX-HpH RPLC-LpH RPLC to identify more than 5000 proteins in mouse embryonic fibroblast cells [51]. Besides, several combinations of 3D separation methods have been established, including isoelectric focusing-SCX-RPLC [52], RPLC-strong anion exchange-RPLC [53], electrostatic repulsion hydrophilic interaction chromatography-HpH RPLC-LpH RPLC [54], and SCX-RPLC-CZE [55]. Recently, Spicer et al. developed a 3D system consisting of three consecutive RPLC, which identified more than 14,000 proteins across 126 fractions [56].

In this study, in-depth proteomic profiling of *E. huxleyi* (CCMP371) was performed using a 3D-LC system. The 3D-LC strategy consisted of SCX and HpH RPLC fractionation, followed by LpH RPLC separation and tandem mass spectrometry (MS/MS) analysis. Seventy SCX-HpH RPLC fractions were generated from *E. huxleyi* proteome digest. Peptide and protein identification was performed using Trans-Proteomics Pipeline (TPP). The physicochemical properties of the identified peptides and proteins were evaluated. In addition, the identified proteins were used to define functional classifications based on gene ontology (GO) and Kyoto Encyclopedia of Gene and Genomes (KEGG) pathway through ClueGO.

## 2. Results

### 2.1. Design of an Off-line 3D-LC (SCX-HpH RPLC-LpH RPLC) System

**Figure 1:**
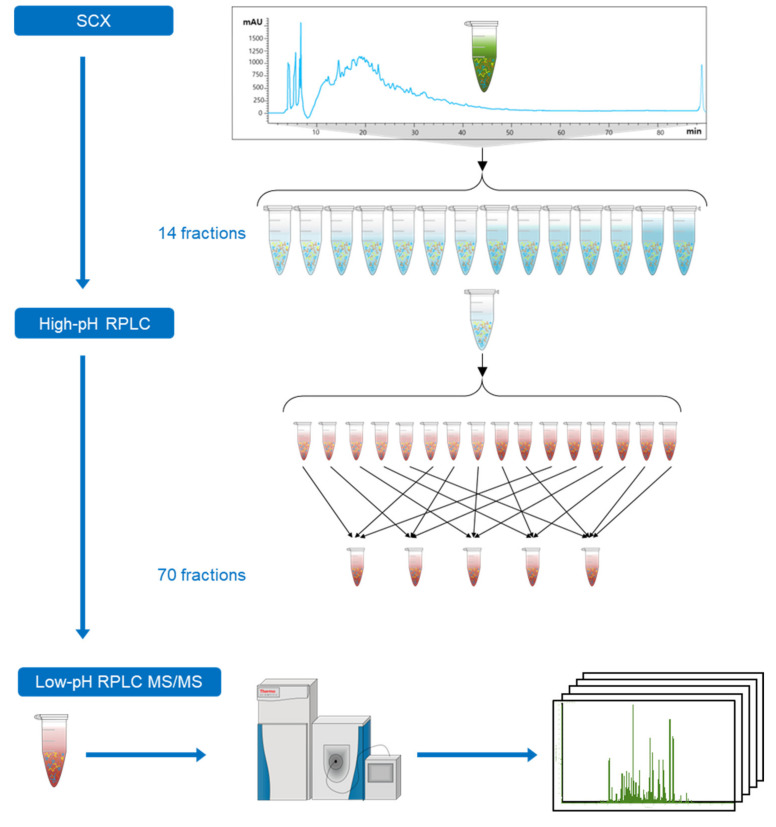
Workflow of proteomic analysis of *Emiliania huxleyi (E. huxleyi)* using three-dimensional liquid chromatography (3D-LC) system. The proteome digest of *E. huxleyi* (CCMP371) cell lysates was fractionated into 14 strong cation exchange (SCX) fractions. Each of them was further separated into 16 fractions using high-pH reversed-phase liquid chromatography (HpH RPLC). A set of 16 fractions generated from one SCX fraction was concatenated into five final fractions (1-6-11, 2-7-12, 3-8-13, 4-9-14, and 5-10-15-16). Overall, 70 fractions were generated from the original proteome digest of *E. huxleyi* cell lysates for liquid chromatography -tandem mass spectrometry (LC-MS/MS) analyses.

In this study, an off-line 3D-LC separation system was coupled with the Q Exactive™ Hybrid Quadrupole-Orbitrap mass spectrometer to identify a large number of proteins from *E. huxleyi* cell lysates (Figure 1). The proteome digest of *E. huxleyi* cell lysates was first fractionated into 14 fractions (C to P) using SCX. After that, each of them was further separated into 16 fractions using HpH RPLC. A set of 16 fractions generated from one SCX fraction was concatenated into five final fractions (1-6-11, 2-7-12, 3-8-13, 4-9-14, and 5-10-15-16). Combination of fractions with different hydrophobicity in the HpH RP dimension could reduce the LpH RPLC–MS/MS analysis time without a significant reduction in protein identification [43]. Overall, 70 fractions were generated from the original proteome digest of *E. huxleyi* cell lysates for LC–MS/MS analyses.

### 2.2. Proteomic Analysis of E. huxleyi Using 3D-LC System

#### 2.2.1. Identification of Peptide and Protein Group

**Figure 2:**
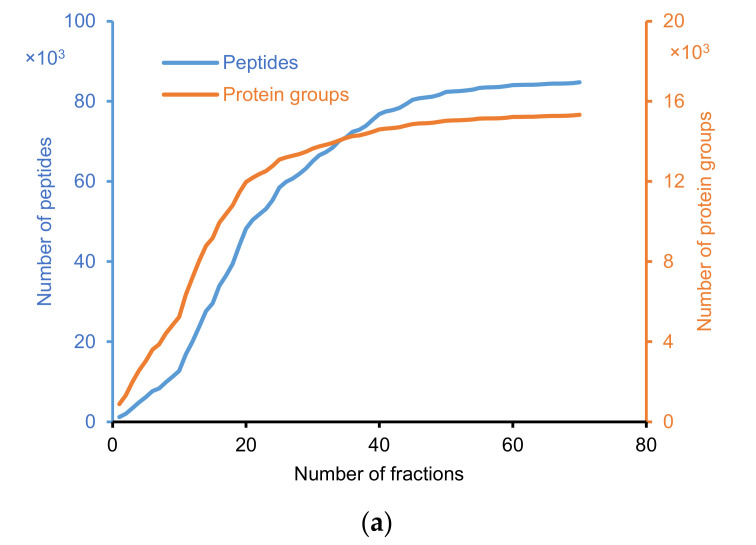

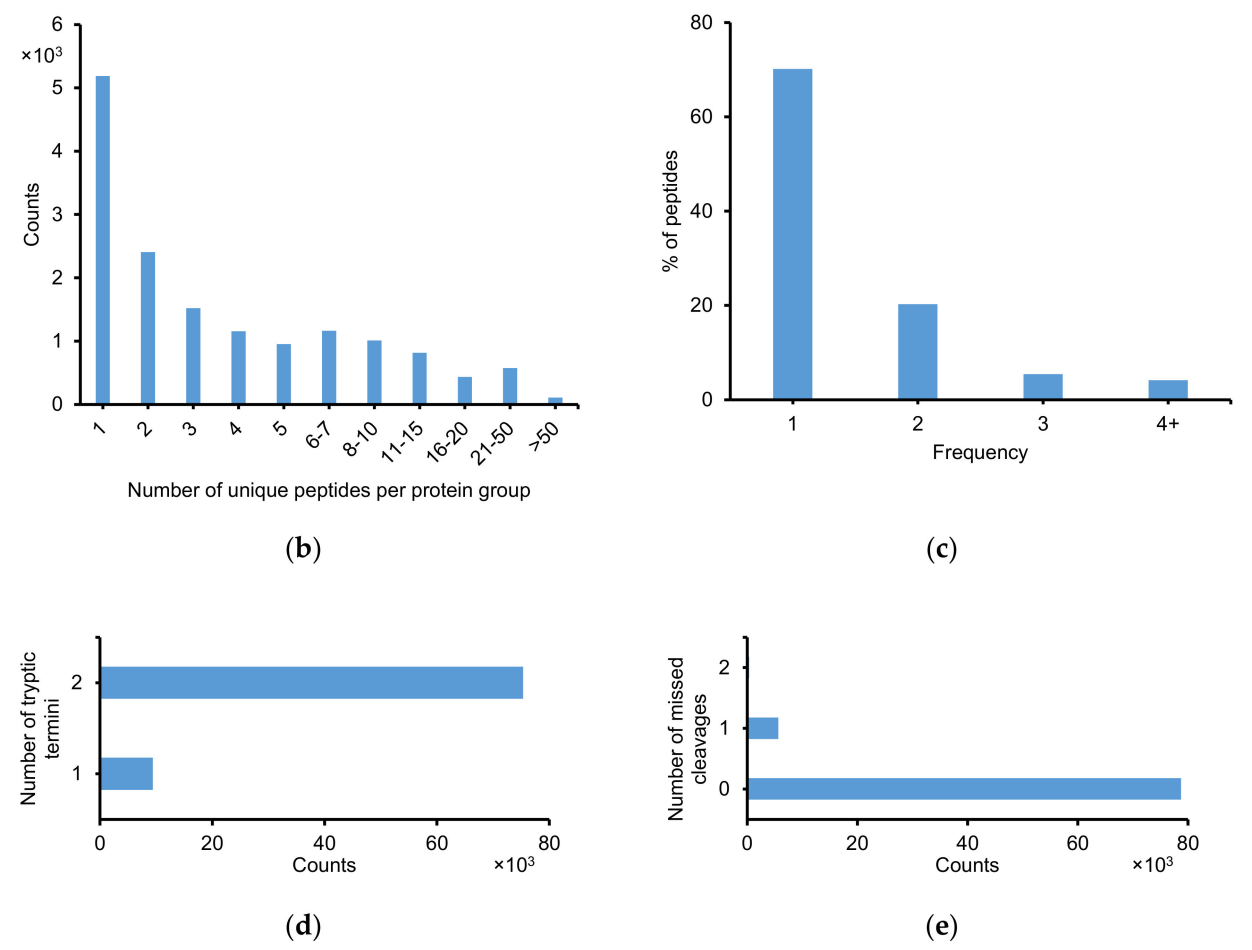
Summary of the results from proteomic profiling of *E. huxleyi* using 3D-LC system. (**a**) The accumulated numbers of identified peptides and protein groups (single hit included) versus the number of fractions. (**b**) Number of unique peptides per protein group. (**c**) Distribution of peptides by frequencies in 70 fractions. (**d**) Number of tryptic termini in identified peptides. (**e**) Number of missed cleavages in identified peptides.

The proteome of *E. huxleyi* was searched against a Uniprot database using TPP. After MS/MS search with Comet, the data were further analyzed with PeptideProphet and ProteinProphet. The list of peptides identified by PeptideProphet at a false discovery rate (FDR) ≤0.01 is shown in Table S1. The term “protein” means a distinct protein, whereas the “protein group” indicates indistinguishable proteins (with shared peptides) grouped by ProteinProphet [57]. For simplification, “protein group” was used for both proteins and protein groups. Figure 2 presents the results of *E. huxleyi* proteomic profiling using the developed 3D-LC system. In total, 84,753 unique peptides and 15,331 protein groups (single hit included) were identified (Figure 2a). From 35,707 protein entries in the database, 43% of protein coverage was obtained (single hit included). The identification of peptides and protein groups gradually increased with the number of fractions. However, the increase rate reduced in the last fractions (~50–70), most likely due to the fraction-to-fraction overlap of the peptides, although this overlap was relatively small (Figure 2c). Figure 2b exhibits the distribution of protein groups by the number of unique peptides. When single hits were excluded, the number of protein groups was 10,145. It can be observed that over 90% of the identified peptides constrained within one to two fractions, and approximately 96% of them spanned in one to three fractions (Figure 2c). This finding demonstrates minimal fraction-to-fraction overlap, as well as the sufficient power of the SCX-HpH RPLC fractionation. The effectiveness of tryptic digestion was demonstrated in Figure 2d,e. Nearly 90% of the peptides were identified with two tryptic termini, whereas approximately 7% of the peptides contained one to two missing cleavages.

#### 2.2.2. Physicochemical Properties of Identified Peptides

**Figure 3:**
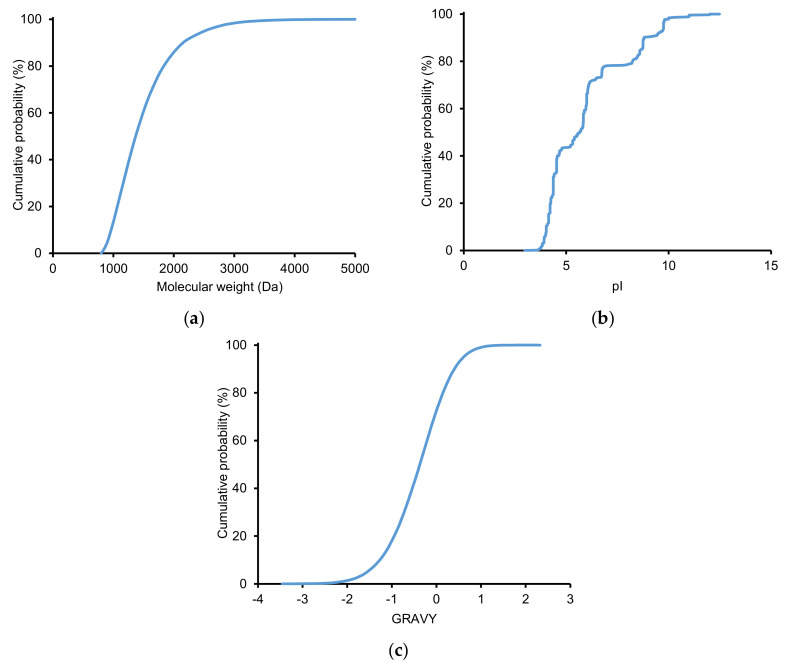
Cumulative distribution of (**a**) molecular weight, (**b**) isoelectric point (pI), and (**c**) the grand average of hydropathy (GRAVY) value of identified peptides. GRAVY values of peptides were calculated using the GRAVY calculator (http://gravy-calculator.de/); negative GRAVY values signify hydrophilicity, and positive values indicate hydrophobicity.

The physicochemical properties of the identified peptides from the 3D-LC system were also evaluated. Retention time (Rt), molecular weight (MW), and isoelectric point (pI) of peptides were obtained using TPP. The pI value is the pH where the peptide carries no net charge. At a pH below pI, the peptide has a positive charge and vice versa. The grand average of hydropathy (GRAVY) values of peptides were calculated using the GRAVY calculator (http://gravy-calculator.de/) [55]. Negative GRAVY values signify hydrophilicity, and positive values indicate hydrophobicity. Figure 3 presents the cumulative distribution of MW, pI, and GRAVY of identified peptides. The majority of the identified peptides have MW in the range 800–2000 Da (86%) and GRAVY <0 (72%). Approximately 78% of the peptides have pI in the range 3.5–7.0. The distribution of pI is similar to a previous report using SCX-LpH RPLC [42]. We constructed various two-dimensional graphs to elucidate the relationship among different parameters of the identified peptides (Figure S1). We can observe that the majority of high MW peptides with MW >2000 Da was eluted at a later time in LpH RPLC (Rt of 60 to 130 min) (Figure S1a, Supplementary Materials). They mostly had GRAVY of −1.5~+1 (Figure S1d) and pI of 3 to 7 (Figure S1e). Acidic peptides seemed to appear throughout LpH RPLC running time, whereas basic peptides, particularly those with pI >11, mostly presented at 30 to 90 min (Figure S1b). As shown in Figure S1c, highly hydrophilic peptides were likely associated with a short Rt, whereas hydrophobic peptides were eluted throughout the LpH RPLC running time. GRAVY and pI do not show any distinct relationship (Figure S1f).

#### 2.2.3. Effects of Strong Cation Exchange (SCX) on Peptide Separation

The first separation step (SCX) generated 14 fractions (which were named as C–P) corresponding to the increase in the KCl concentration of the elution buffer. The identified peptides were grouped by SCX fractions (overlap peptides were kept in each SCX fraction), and various box-plots were constructed to elucidate the effect of SCX (i.e., KCl concentration) on the separation of peptides (Figure S2). It is evident that SCX did not considerably separate the peptides regarding their MW and GRAVY (Figure S2a,d). However, as shown in Figure S2b, peptides with low pI values were eluted at a low salt concentration and vice versa. This finding is in agreement with the theoretical characteristics of SCX [41]. In SCX, electrostatic interaction mainly affects the retention of peptides, whereas hydrophobic interactions only play a modest role [58]. In this study, SCX was operated at a low pH (2.7) to reduce the dissociation of carboxylic groups of peptides and thereby promote interactions between the sulfonate groups of SCX resins and the protonated basic amino acid residues [40]. Under pH 2.7, peptides with pI >2.7 were positively charged and bound to the aliphatic sulfonic acid groups of SCX resins. The peptides with higher pI values bound stronger to the stationary phase than those with lower pI values [41]. A salt gradient (KCl) was used to separate the peptides depending on their charge. Peptides with pI values closer to 2.7 would be eluted first when applying a lower ionic strength. As the salt concentration was higher, peptides with higher pI would be eluted. Besides, Figure S2c shows that peptides corresponding to a low salt strength in SCX (eluted at a low Rt) seemed to have higher Rt in LpH RPLC. It suggests that SCX and LpH RPLC have a high orthogonality.

#### 2.2.4. Protein Identification Using ProteinProphet

**Figure 4:**
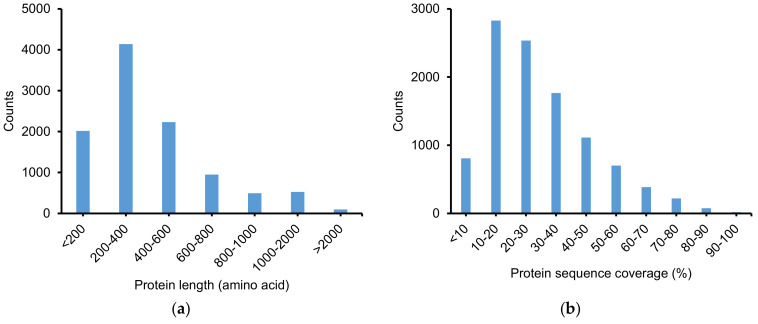
Distribution of identified proteins groups according to (**a**) protein length and (**b**) protein sequence coverage.

The list of protein groups identified by ProteinProphet at an FDR ≤0.01 is shown in Table S2. A total of 10,448 protein groups were identified with at least two unique peptides. Figure 4 exhibits the distribution of the protein groups by their length and protein sequence coverage (%). Most of the protein groups had <600 amino acids (80%). Additionally, about 79% of the protein group had a sequence coverage percentage of 10 to 50%. Among more than 10,000 identified proteins groups, only 1770 protein groups (~17%) have been characterized, whereas the majority were defined as uncharacterized proteins in the Uniprot database.

### 2.3. Gene Ontology (GO) and Kyoto Encyclopedia of Gene and Genomes (KEGG)

**Figure 5:**
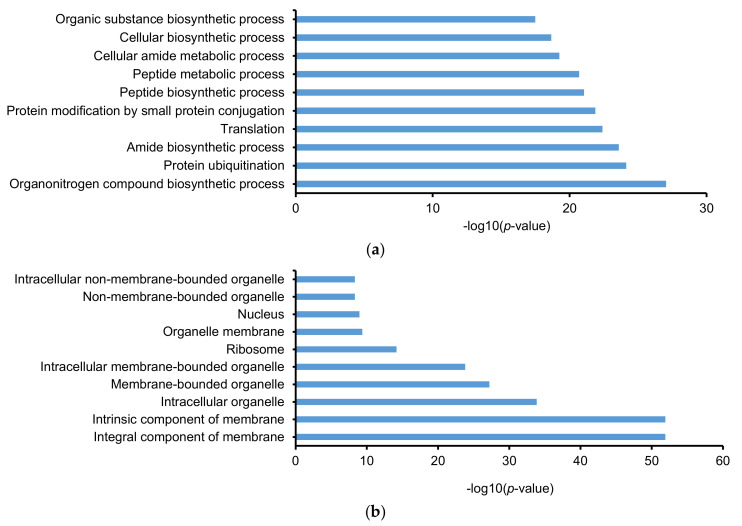

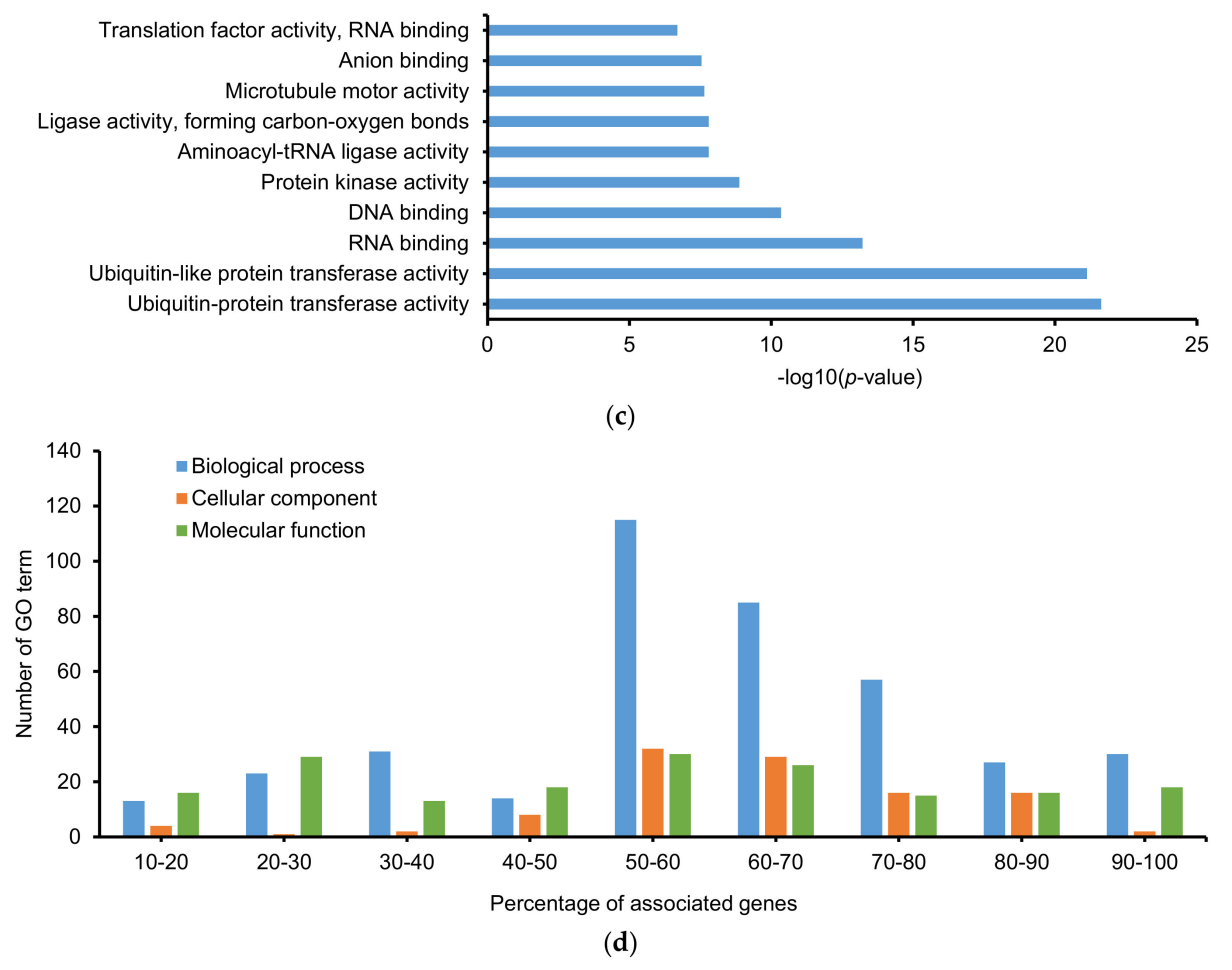
Gene ontology (GO) analysis of identified protein groups from *E. huxleyi* (CCMP371) using ClueGO. Top 10 of (**a**) biological processes, (**b**) cellular components, and (**c**) molecular functions regarding −log10(*p*-value). (**d**) Summary of GO term number according to % associated genes.

ClueGO add-in (Cytoscape) was used to categorize the identified proteins/protein groups based on GO and KEGG pathways. The database of *E. huxleyi* contained 2802 biological processes (9885 genes), 509 cellular components (8596 genes), 1452 molecular functions (12,470 genes), and 104 KEGG pathways (3259 genes), which was updated on 31 October, 2019. In total, 1454 biological processes (51.9%), 235 cellular components (46.2%), 678 molecular functions (46.7%), and 100 KEGG pathways (96.2%) were found. After filtering with a *p*-value of ≤0.05, 15 KEGG pathways were defined and listed in Table 1. The percentages of associated genes vary from 21.1 to 83.9%. Some KEGG pathways were identified with high percentages of associated genes, such as basal transcription factors, citrate cycle (TCA cycle), proteasome, spliceosome, mRNA surveillance pathway, protein processing in endoplasmic reticulum, and amino sugar and nucleotide sugar metabolism (≥67%). In addition, after filtering with a *p*-value of ≤0.05, the identified protein groups are involved in 395 biological processes, 110 cellular components, and 181 molecular functions (Table S3). Top 10 GO terms with highest −log10(*p*-value) were presented in Figure 5a–c. The top 10 biological processes are biosynthetic processes, metabolic processes, translation, protein modification, and protein ubiquitination. The top 10 cellular components are components of membrane, nucleus, ribosome, and different organelles. The top 10 molecular functions are transferase, ligase, and kinase activity, and different binding functions. Figure 5d classifies GO terms regarding the percentage of associated genes. It is evident that most of the GO terms have 50 to 80% associated genes.

## 3. Discussion

Various 2D-LC and few 3D-LC separation systems have been applied to proteomic researches for decades to solve the under-sampling issue (i.e., the complexity of peptide mixtures is too high to be fully detected by mass spectrometry). The developed 3D-LC system can minimize this limitation by increasing peptide and protein identification. This system is based on the high orthogonality between SCX and RPLCs as well as the efficacy of two RPLC with an extreme pH difference [33,44,45]. SCX was performed as the first-dimensional separation to separate peptides based on their charges [55,59]. Then, peptides were fractionated by HpH RPLC with respect to their hydrophobicity [60]. Lastly, LC-MS/MS with a low-pH solvent was performed as the third separation. The orthogonality between HpH RPLC and LpH RPLC was due to the pH of mobile phases, as previously reported. The pH affected the net charge of peptides according to the pKa values of their ionizable amino acid groups. Thus, basic peptides were more retained at high pH, while acidic peptides were retained at low pH [43,44]. The developed sequential 3D-LC platform was performed in an off-line manner to overcome the limitation of small loading sample amounts, an obvious drawback of online multi-dimensional separation approaches [29]. Besides, an off-line fractionation method could dramatically improve separation power, since it is highly flexible and easy to manipulate between separations, and thus each dimensional separation can be optimized by varying buffer and elution conditions [29,48,61]. Another improvement of our 3D-LC system is the arrangement of the three different LC systems. SCX has a high sample loading capacity serving as the first separation. The desalting step is often required after SCX to remove salts in samples, which can reduce sensitivity or cause blockage in the LC-MS/MS column [62]. However, the C18 column of HpH RPLC following SCX can serve as the desalting column for SCX-fractions. Therefore, this 3D-LC system reduces additional steps after SCX, as previously reported by Betancourt et al. [51]. This procedure, however, was complicated due to additional steps (acylation of amino groups before SCX fractionation and regeneration of amino groups after that). In addition, the concatenation of fractions in the second separation dimension reduces the total number of SCX-HpH RPLC from 224 to 70, which is quite moderate for LpH RPLC–MS/MS analysis [43]. Seventy fractions were analyzed by the LpH RPLC–MS/MS in 175 h (150 min per analysis). It is relatively long (~1 week), as an inevitable result of a large fraction number in a 3D-LC approach, but it is necessary for a deep proteomic profiling study [56]. Depending on the biological question of each study, the number of SCX-HpH RPLC fractions may be adjusted, and thus, the MS/MS analysis time can also be altered. In comparative proteomics, due to the increase in the number of samples, the analysis time may increase substantially with this 3D-LC method. However, a labeling method can allow the simultaneous process of many samples, and thereby, the MS/MS analysis time remains unchanged [26].

Some previous studies investigated the proteome of *E. huxleyi*. A shotgun proteomic analysis identified 99 proteins from *E. huxleyi* (NZEH) [12]. Another study used tandem mass-spectrometry with isobaric tagging (iTRAQ) to reveal changes in protein abundance between normal and high CO_2_ conditions. However, due to the limited number of quantified proteins, acclimation mechanisms tolerating high CO_2_ amount remained unrevealed [13]. A study of McKew et al. investigated acclimation of *E. huxleyi* to nutrient limitation and found the marked increases in the abundance of some proteins belonging to inorganic nutrient transport and the internal remobilization of N and P compounds [15]. In this study, utilizing the 3D-LC system for the in-depth profiling of *E. huxleyi* (CCMP371), we identified more than 84,000 unique peptides and above 10,000 protein groups (with at least two unique peptides) at an FDR of ≤0.01. The 3D-LC system was not evaluated regarding the reproducibility. It could be carried out by dividing the proteome digest into two to three parts after digestion and simultaneously performing the peptide fractionation and LC-MS/MS analysis. Generally, 2D- and 3D-LC systems suffer low reproducibility in quantification. However, these issues have been solved in recent studies using different labeling methods, which allow the simultaneous handling of many samples [26]. This 3D-LC approach may also present a low reproducibility. Nevertheless, it could be used in comparative proteomic studies to reveal changes in protein levels and define related mechanisms under different conditions. Some labeling methods may necessarily be incorporated to allow the implementation of many samples under identical conditions, such as metabolic labeling [63], stable isotope labeling using amino acids in cell culture (SILAC) [64], tandem mass tags (TMT) [65], and iTRAQ [66].

Some of the identified protein groups have been defined as playing critical roles in various processes of *E. huxleyi*. For example, proteins involved in carbon transport, pH homeostasis, and biomineralization were found, such as carbonic anhydrases, vacuolar H^+^ ATPase, plasma membrane ATPase, H^+^/Ca^2+^ exchanger, Na^+^/Ca^2+^-K^+^ exchanger, Ca^2+^ ATPase, and cation/Ca^2+^ exchanger. The identification of these proteins in a proteomic study could allow a direct measurement of their abundance or activity, giving a better insight into the cellular changes under different conditions compared to the measurement of transcripts [67]. Carbonic anhydrases are able to catalyze the interconversion water and CO_2_ with H^+^ and bicarbonate [12]. The regulation of carbonic anhydrases is linked to biomineralization in coccolithophores [68]. Vacuolar H^+^ ATPase and clathrin are related to calcifying vesicle membranes. Some of the ATPase subunits identified in this study may be a part of a complex in *E. huxleyi* membranes, which maintains an alkali pH together with the formation of CaCO_3_ [12]. Clathrin-coated vesicles contain vacuolar H^+^ ATPase as previously reported [69]. These vesicles participate in protein and lipid transportation, as well as some membrane and trans-Golgi network trafficking pathways [70]. In *E. huxleyi*, coccolith formation occurs in Golgi-derived coccolith vesicles, and clathrin-coated vesicles containing vacuolar H^+^ ATPase play a role in the alkalization for coccolith formation [12,71]. Under ocean acidification, calcifying marine organisms tend to shift the interconversion to increase H^+^ and bicarbonate concentration. A proteomic approach may help to reveal proteome changes and related mechanisms. In *E. huxleyi* (strain NZEH), under high CO_2_ level, abundances of ribosomal proteins (30S ribosomal protein S7) and histones (H2A, H3, and H4) decreased, indicating a reduction in DNA and chromatin synthesis [13]. Besides calcification, biomineralization also includes silicification. In some coccolithophores, diatom-like silicon transporters are present for silicification, as Si also plays a crucial role in the formation of calcite coccoliths. However, *E. huxleyi* is one of the species that lack the requirement for Si [72]. The proteomic results in our study also confirmed the absence of any Si-related proteins in *E. huxleyi*. In cellular component, adaptor protein (AP)-type membrane coat adaptor complex was identified, specifically subunits of AP1 and AP2 complexes. Five different AP complexes (AP1-5) relate to biomineralization by transporting vesicles intracellularly. It was reported that a partial or complete loss of AP3 and AP5 in the gene-level has occurred in three coccolithophores (*Isochrysis galbana*, *Gephyrocapsa oceanica*, and *E. huxleyi* strain Van 556, 92A, EH2, and CCMP1516) as a consequence of evolutionary events [73]. Similarly, AP3 and AP5 were not identified in *E. huxleyi* (strain CCMP371) in this study, suggesting their loss or low abundances.

Various identified proteins groups are involved in photosynthesis (77), photosynthesis, light-harvesting (60), and photosynthesis, light reaction (68). In addition, chloroplast (78 protein groups), chloroplast thylakoid (62 protein groups), and chloroplast thylakoid membrane (62 protein groups) were defined, which are essential cellular components that mediate the effect of light on calcification in *E. huxleyi* [74]. A previous proteomic study found 49 light-harvesting complex proteins and 12 photosynthetic electron transfer chain proteins. The abundances of these proteins varied depending on suboptimal or supra-optimal light. Proteins in photosystem I and II increased their abundance in low light, whereas light-harvesting fucoxanthin–chlorophyll proteins and photoprotective LI818 proteins up-regulated in high light [14]. Some transport processes are involved in the transport of protein, peptide, amine, nitrogen compound, organic substance, Golgi vesicle, xenobiotic, cation, anion, ammonium, carbohydrate, metal ion, RNA, and nucleic acid. Upon changes in environmental conditions, the relevant transport proteins may show abundance changes. For example, during periods of phosphate starvation, proteins involved in phosphorus transport are more abundant and vice versa [15,75]. Similarly, upon the limitation of nitrogen-containing compounds, various nitrogen transporters are up-regulated [15]. In addition, the nitrogen starvation also leads to the up-regulation of glutamine synthetase, a protein frequently used by marine bacterioplankton to dissolve organic or inorganic nitrogen [76,77]. In this study, glutamine synthetase was also detected, which related to the assimilation of ammonium into amino acids.

Many metabolic processes relating to peptide, amide, carboxylic acid, organic acid, amino acid, cellular nitrogen compound, cellular carbohydrate, RNA, ribose phosphate, ribonucleotide, nucleoside phosphate, heme, and glutathione were identified. Biosynthesis of nucleoside, ribonucleoside, peptidyl-diphthamide, DNA, glycosyl compound, quinone, and inositol phosphate were also listed in biological processes. These results can favor further studies to identify some molecules in *E. huxleyi*, such as primary/secondary metabolites and bioactive molecules that can be potentially applied in pharmaceutical, cosmeceutical, and nutraceutical, like other marine organisms [78]. The glycosyl compound-quinone biosynthetic process and glutathione metabolic process were defined. They hold potential for the discovery of biopharmaceuticals and antioxidant cosmeceuticals. Previously, several secondary metabolite molecules from marine cyanobacteria were defined as potential anticancer biomolecules [79]. Phlorotannins from brown algae have hypopigmentation effects, which can be applied in the cosmetic industry. Docosahexaenoic acid-rich oils derived from *Thraustochytrids* are currently available on the market as a dietary supplement [80]. In addition, various molecular functions relating to binding (proteins, l-ascorbic acid, vitamin B6, and iron ion) and enzyme activities (transferase, kinase, peptidase, lipase, and phosphatase) were identified. Vitamins and minerals in *E. huxleyi* can possibly be exploited as a useful source of food ingredients or nutraceutical supplementary. This study only revealed the proteome of *E. huxleyi*, whereas the metabolome was not accessed, and thus, many critical features relating to metabolites in this species remained unknown. Several studies have performed an integrated metabolomics-proteomics, which could show a better landscape of the marine plankton [81,82]. Integrating “-omics” studies (e.g., genomics, proteomics, transcriptomics, metabolomics, lipidomics, and glycomics) will enable the investigation of the impacts of ecological interactions on organism physiology as well as evaluation of physiological and biochemical properties relating to human health for future applications [83,84].

## 4. Materials and Methods

### 4.1. Materials

*E. huxleyi* strain CCMP371 was purchased from the Provasoli-Guillard National Center for Marine Algae and Microbiota (NCMA, East Boothbay, ME, USA). Tris (2-carboxyethyl)phosphine (TCEP) was supplied by Thermo Fisher Scientific (Rockford, IL, USA). Formic acid, iodoacetamide (IAA), ammonium bicarbonate (ABC), and potassium chloride were obtained from Sigma-Aldrich (St. Louis, MO, USA). Trypsin was received from Promega (Madison, WI, USA). HPLC-grade water and acetonitrile were purchased from J.T. Baker (Phillipsburg, NJ, USA). All the chemicals were used as received without further purification.

### 4.2. Cell Culture

*E. huxleyi* (CCMP371) cells were cultured in 500 mL-Erlenmeyer flasks under 12-h light/dark cycles at 20 °C. The media contained sterile artificial seawater enriched with nitrates, phosphates, trace metals, vitamins at f/2 concentration, and selenium [85,86]. The irradiance of fluorescent lamps was set at 50 μmol m^−2^ s^−1^.

### 4.3. Sample Preparation

#### 4.3.1. Protein Extraction

The cell pellets were placed in Maintainor^®^ Tissue cards and stabilized at 95 °C in Stabilizor^TM^ T1 (Denator, Gothenburg, Sweden) for denaturing. The samples were then loaded into pre-chilled TT1 tissue TUBE^TM^ (Covaris, Woburn, MA, USA), frozen in liquid nitrogen followed by pulverization using CryoPrep^®^ (Covaris). Proteins were extracted in lysis buffer (8 M urea and 0.1 M Tris-HCl, pH 8.5) under sonication (12 min, 18 °C, Covaris). Four volumes of acetone (−20 °C) were added to the sample, followed by incubation at −20 °C overnight for protein precipitation. After centrifugation (4000 rpm, 4 °C, 10 min, Centrifuge 5810 R, Eppendorf, Hamburg, Germany), the supernatant was discarded, and the protein pellets were dried in a ScanSpeed 40 centrifugal evaporator (1800 rpm, 3 h, Labogene, Lillerød, Denmark). The purified proteins were re-suspended in the lysis buffer followed by protein quantification using the Pierce BCA Protein Assay kit (Thermo Fisher Scientific, Rockford, IL, USA).

#### 4.3.2. Automated Filter-Aided Sample Preparation (FASP)

A liquid handling robotic system (Agilent Technologies, Santa Clara, CA, USA) controlled by VWorks software was used to perform the automated FASP [87,88]. Multiscreen Vacuum Manifold^™^ (Millipore, Billerica, MA, USA) was used to supply vacuum during sample preparation. In brief, protein samples were first loaded onto 50 wells of a 96-well plate (AcroPrep Advance Filter Plates, 350 µL, Omega 30K MWCO) with 100 µg protein/well. Proteins were reduced with 100 µL of 5 mM TCEP (37 °C, 30 min), alkylated with 100 µL of 50 mM IAA (25 °C, 30 min, in the dark), and digested with trypsin at an enzyme: protein ratio of 1:50 (*w/w*) (37 °C, 18 h). The digestion was stopped by reducing the pH of the samples to 2–3 with formic acid. The number of samples was reduced from 50 to 5 by pooling before desalting.

#### 4.3.3. Desalting with Reverse-Phase Solid-Phase Extraction

Sample desalting was conducted using Sep-Pak^®^ Vac 1cc C18 cartridge (Waters, Milford, MA, USA). The cartridge was washed with 1 mL of methanol followed by 1 mL of solvent A_1_ (0.1% formic acid in water), equilibrated with 1 mL of solvent B_1_ (0.1% formic acid in acetonitrile: water (80:20, *v/v*)), and then washed with 1 mL of solvent A_1_. The digested samples were loaded onto the column and washed with 500 μL of solvent A_1_. The peptides were eluted with 1 mL of solvent B_1_ and subsequently dried in the ScanSpeed 40 centrifugal evaporator (1800 rpm, 3 h). Samples were dissolved in solvent A_1_ and pooled to one prior to fractionation.

#### 4.3.4. SCX Fractionation

The peptide mixtures were fractionated by SCX chromatography using an HPLC system consisting of a vacuum degasser, a binary pump, an autosampler (Agilent series 1200, Agilent Technologies), and a UV detector (Agilent series 1100, Agilent Technologies). Sample separation was carried out on a Polysulfoethyl A^TM^ column (4.6 × 250 mm, 5 μm particles, 100-Å pores, PolyLC, Columbia, MD, USA) using a flow rate of 0.5 mL/min. The mobile phase solvent consisted of A_2_ (10 mM KH_2_PO_4_ in acetonitrile: water (20:80, *v/v*), pH 2.7) and B_2_ (10 mM KH_2_PO_4_ and 0.6 M KCl in acetonitrile: water (20:80, *v/v*), pH 2.7). The column was equilibrated with solvent A_2_ for 30 min. The gradient was applied as follows for solvent B_2_: 0–10% for 2 min, 10–80% for 68 min, 80–100% for 5 min, and holding at 100% for 15 min. The column was re-equilibrated using solvent A_2_ for 30 min and stored at −4 °C. The eluting peptides were monitored at 214 nm and collected automatically into tubes at 3 min intervals. The eluted samples were dried in the ScanSpeed 40 centrifugal evaporator (1800 rpm, 3 h). According to the peptide concentration of each fraction, some fractions were unchanged, whereas several two to three adjacent fractions were pooled to make 14 SCX-fractions (C to P).

#### 4.3.5. HpH RPLC Fractionation

HpH RPLC was carried out using Sep-Pak^®^ Vac 1cc tC18 cartridges (Waters, Milford, MA, USA). The column was washed in the following steps: 1 mL of 100% methanol, 1 mL of solvent A_1_ (0.1% formic acid in water), 1 mL of solvent B_1_ (0.1% formic acid in acetonitrile: water (80:20, *v/v*)), and 1 mL of solvent A_1_. Peptide mixtures in each SCX fraction were loaded onto the column and washed with 0.5 mL of solvent A_1_. Peptides were eluted with 16 eluting solvents containing 0.1% trimethylamine in different acetonitrile: water mixtures. The amount of acetonitrile was varied as follows: 2.5, 5, 6.25, 7.5, 8.75, 10, 11.25, 12.5, 13.75, 15, 16.25, 17.5, 18.75, 20, 35, and 50% (*v/v*). Sixteen fractions (numbered from 1 to 16) were pooled to make 5 HpH RPLC fractions (1-6-11, 2-7-12, 3-8-13, 4-9-14, and 5-10-15-16) [43]. The obtained fractions were labeled, e.g., C1, C2, C3, C4, and C5 were the five HpH RPLC fractions derived from SCX-fraction C. The samples were then dried in the ScanSpeed 40 centrifugal evaporator (1800 rpm, 3 h).

### 4.4. LC–MS/MS Analysis

The dried peptides were reconstituted in 50 μL of solvent A_1_ (0.1% formic acid in water) and injected (1 μg) for analysis. The LC-MS/MS system consisted of a Dionex Ultimate 3000 HPLC coupled with a Q Exactive™ Hybrid Quadrupole-Orbitrap mass spectrometer (Thermo Fisher Scientific, Rockford, IL, USA). The peptides were loaded onto an Acclaim™ PepMap™ 100 C18 nano-trap column (75 μm × 2 cm, 3 μm particles, 100-Å pores, Thermo Fisher Scientific) using solvent A_1_ (0.1% formic acid in water) at a flow rate of 2.5 μL/min for 5 min. Then, the peptides were separated in an Acclaim™ PepMap™ C18 100A RSLC nano-column (75 μm × 50 cm, 2 μm particles, 100-Å pores, Thermo Fisher Scientific) at a flow rate of 300 nL/min. The mobile phase solvent consisted of A_3_ (0.1% formic acid in water) and B_3_ (0.1% formic acid in acetonitrile: water (90:10, *v/v*)). The gradient was set up as follows for solvent B_3_: equilibration at 4% for 10 min, 4–40% for 110 min, 40–96% for 0.1 min, holding at 96% for 9.9 min, 96–4% for 0.1 min, and holding at 4% for 19.9 min for re-equilibration of the column. The following parameters were used: spray voltage, 2.2 kV; capillary temperature, 320 °C; isolation width, ± 2 *m/z*; scan range, 400–2000 *m/z*; resolution in full-MS scans, 70,000; and resolution in MS/MS scans at 200 *m/z*, 17,500. The MS was operated using a data-dependent acquisition method. Top ten precursor ions with the highest intensity were isolated in the quadrupole and fragmented by the higher-energy collisional dissociation with 27% normalized collisional energy. Dynamic exclusion was set at 20 s to minimize the repeated analyses of the same abundant precursor ions.

### 4.5. Data Analysis

The proteomics data have been deposited to the ProteomeXchange Consortium via the PRIDE partner repository [89], with the dataset identifier PXD018511. Raw MS/MS data files were converted to. mzXML format by MSConvert. Comet (version 2017.01 rev.0) was used to search MS/MS spectra against a database of *E. huxleyi* (CCMP371) obtained from Uniprot. The draft release of *E. huxleyi* genome assembly version 1.0 includes a total of 39,126 predicted gene models and functional annotations using the JGI annotation pipeline [11]. The best gene model at each locus, including diploid alleles, was the filtered set selected by the JGI annotation pipeline. The following parameters were set for the search: maximum of two missed cleavages with trypsin; semitryptic cleavage; 10 ppm and 0.02 Da tolerances of precursor ion masses and fragment ion mass, respectively; static carbamidomethylation of cysteine; and variable modifications including methionine oxidation (+15.995 Da) and carbamylation of protein in N-term (+43.0006 Da). The search result files in pepXML format were transported to the TPP version 5.1.0 [90], and PeptideProphet [91] and ProteinProphet [57] were operated. Peptides and proteins were filtered at an FDR of ≤0.01. The data were processed and visualized using MS. Excel 2016. The boxplots were generated using R version 3.6.1. GO [92] and KEGG pathway [93] were categorized using Cytoscape version 3.7.1 (National Institute of General Medical Sciences, MD, USA) via ClueGO version 2.5.4 (Cordeliers Research Center, France) [94]. The GO terms and KEGG pathways were filtered at a *p*-value of ≤0.05.

## 5. Conclusions

In this study, a 3D-LC separation method consisting of SCX-HpH RPLC-LpH RPLC was developed for proteomic profiling of *E. huxleyi*. More than 84,000 unique peptides and 10,000 protein groups were identified from 70 SCX-HpH RPLC fractions. Approximately 700 GO terms and 15 KEGG pathways were defined from the identified protein groups, which relate to various important biological processes, cellular components, and molecular functions of *E. huxleyi*. The identification of *E. huxleyi* proteins in this study will facilitate further studies on this species, particularly those that aim to reveal proteome changes of *E. huxleyi* under different conditions as well as related mechanisms.

## Figures and Tables

**Table 1:** Kyoto Encyclopedia of Gene and Genomes (KEGG) pathways involved in identified protein groups from *E. huxleyi* (CCMP371) using ClueGO.

ID	Description	% Associated Genes	Number of Genes	Term *p*-Value	−log10(*p*-Value)
KEGG:03040	Spliceosome	69.4	120	0.0005	3.3052
KEGG:00531	Glycosaminoglycan degradation	31.0	13	0.0009	3.0608
KEGG:04145	Phagosome	38.0	40	0.0010	3.0222
KEGG:03022	Basal transcription factors	83.9	26	0.0017	2.7735
KEGG:01040	Biosynthesis of unsaturated fatty acids	21.1	4	0.0020	2.6914
KEGG:04141	Protein processing in endoplasmic reticulum	67.7	105	0.0046	2.3355
KEGG:00020	Citrate cycle (TCA cycle)	78.8	26	0.0123	1.9104
KEGG:00330	Arginine and proline metabolism	41.9	26	0.0201	1.6972
KEGG:03050	Proteasome	71.9	41	0.0213	1.6717
KEGG:00430	Taurine and hypotaurine metabolism	23.1	3	0.0217	1.6635
KEGG:04933	AGE-RAGE signaling pathway in diabetic complications	33.3	8	0.0236	1.6280
KEGG:04070	Phosphatidylinositol signaling system	45.3	39	0.0362	1.4411
KEGG:03410	Base excision repair	43.5	27	0.0389	1.4097
KEGG:03015	mRNA surveillance pathway	67.9	55	0.0409	1.3885
KEGG:00520	Amino sugar and nucleotide sugar metabolism	67.0	59	0.0495	1.3052

## Supplementary Materials

The following are available online, Figure S1: Relationship between different parameters of identified peptides, Table S1: List of identified peptides (PeptideProphet) of *Emiliania huxleyi* (CCMP371) in three-dimensional separation method, Table S2: List of identified proteins/protein groups (ProteinProphet) of *Emiliania huxleyi* (CCMP371) in three-dimensional separation method, Table S3: Gene ontology analysis of identified proteins/protein groups from *Emiliania huxleyi* (CCMP371) using ClueGO. The proteomics data have been deposited to the ProteomeXchange Consortium via the PRIDE partner repository with the dataset identifier PXD018511. Reviewers may access it using username reviewer40383@ebi.ac.uk and password wyy3eSj5).
