# Supplementary material for: Proteomic Profiling of Emiliania huxleyi Using a Three-Dimensional Separation Method Combined with Tandem Mass Spectrometry
Source: Molecules. 2020 Jul 2;25(13):3028. doi: 10.3390/molecules25133028 (PMC7411631; doi:10.3390/molecules25133028)
Supplement: Supplementary file 1 [file molecules-25-03028-s001.zip › Supplementary material.docx]

Supplementary material

Proteomic Profiling of *Emiliania huxleyi* Using a Three-dimensional Separation Method

Goyeun Yun ^1,#^, Jong-Moon Park ^1,4,#^, Van-An Duong ^1,#^, Jeong-Hun Mok ^1^, Jongho Jeon ^1^, Onyou Nam ^2^, Joonwon Lee ^3^, EonSeon Jin ^2,*^, and Hookeun Lee ^1,*^

^1^ College of Pharmacy, Gachon University, Incheon 21936, Republic of Korea; ggo1203@hotmail.com (G.Y.); bio4647@naver.com (J.-M.P.); anduong@gachon.ac.kr (V.-A.D.); jeonghunmok@naver.com (J.-H.M.); jeonjh8817@naver.com (J.J.)

^2^ Department of Life Science, Hanyang University, Seoul 04763, Republic of Korea; namonew@naver.com (O.N.)

^3^ College of Letters and Science, University of California Los Angeles, Los Angeles, California 90095, United States; joonwonlee7@gmail.com (J.L.)

^4^ Basilbiotech, Seoul 06621, Republic of Korea; basil@basilbiotech.com (J.-M.P.)

***** Correspondence: hklee@gachon.ac.kr, Tel.: +82-32-820-4927 (H.L.); esjin@hanyang.ac.kr (E.J.)

# These authors contributed equally.

**Table of Content**

**Table S1.** List of identified peptides (PeptideProphet) of *Emiliania huxleyi* (CCMP371) in three-dimensional separation method (MS.Excel file)

**Table S2.** List of identified proteins (ProteinProphet) of *Emiliania huxleyi* (CCMP371) in three-dimensional separation method (MS.Excel file)

**Table S3.** Gene ontology analysis of identified proteins from *Emiliania huxleyi* (CCMP371) using ClueGO: (A) biological process, (B) celluler component, and (C) molecular function (MS.Excel file)

**Figure S1.** Relationship between different parameters of identified peptides. (a) Molecular weight (MW) and retention time. (b) Isoelectric point (pI) and retention time. (c) The grand average of hydropathy (GRAVY) value and retention time. (d) GRAVY and MW. (e) pI and MW. (f) GRAVY and pI.

**Figure S2.** Effect of SCX fractionation on distribution of (a) molecular weight (MW), (b) isoelectric point (pI), (c) RPLC retention time, and (d) the grand average of hydropathy (GRAVY) value of identified peptides.

| **(a)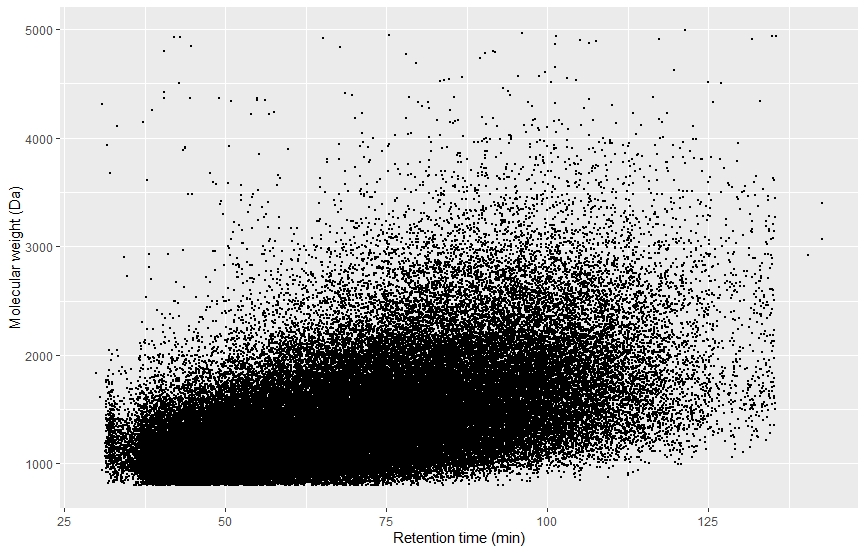** |
| --- |
| **(b)** |
| **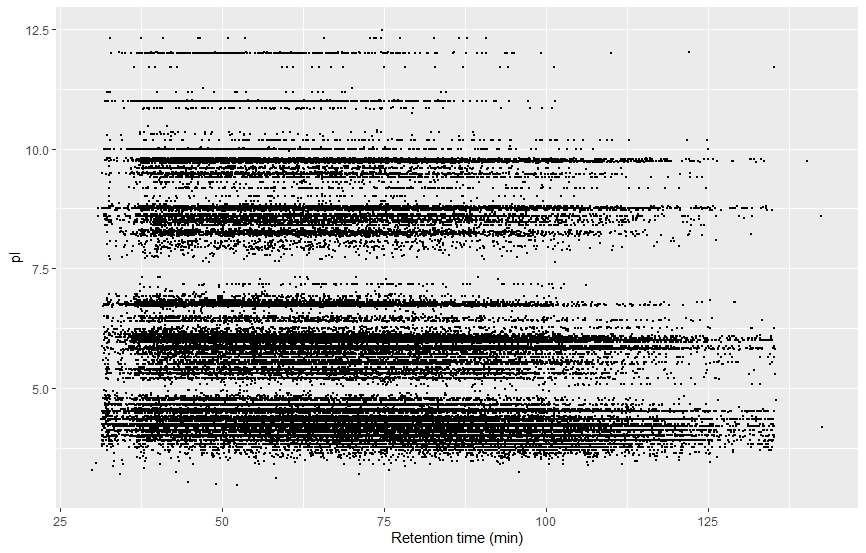** |
| **(c)**  **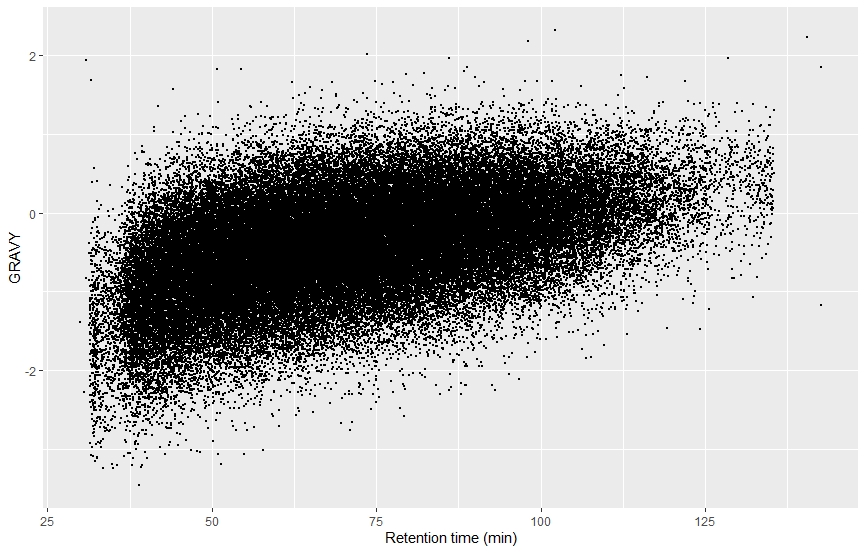** |
| **(d)**  **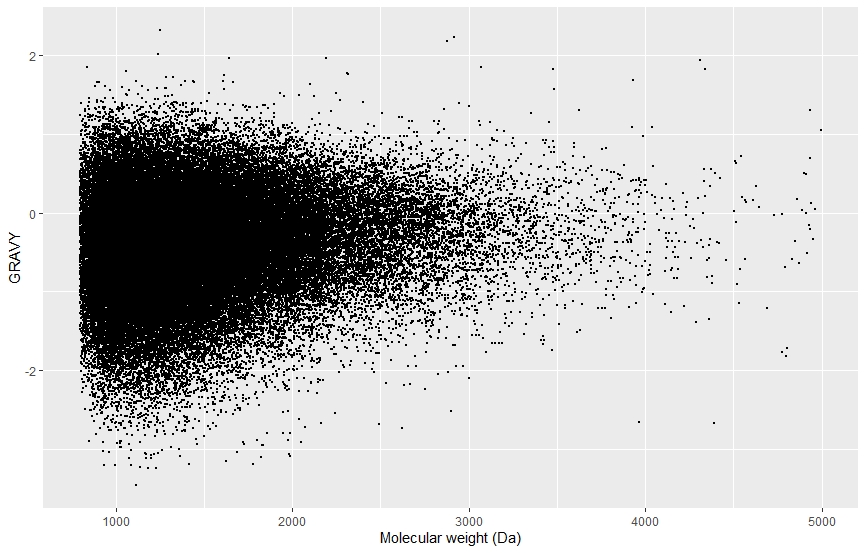** |
|  |
| **(e)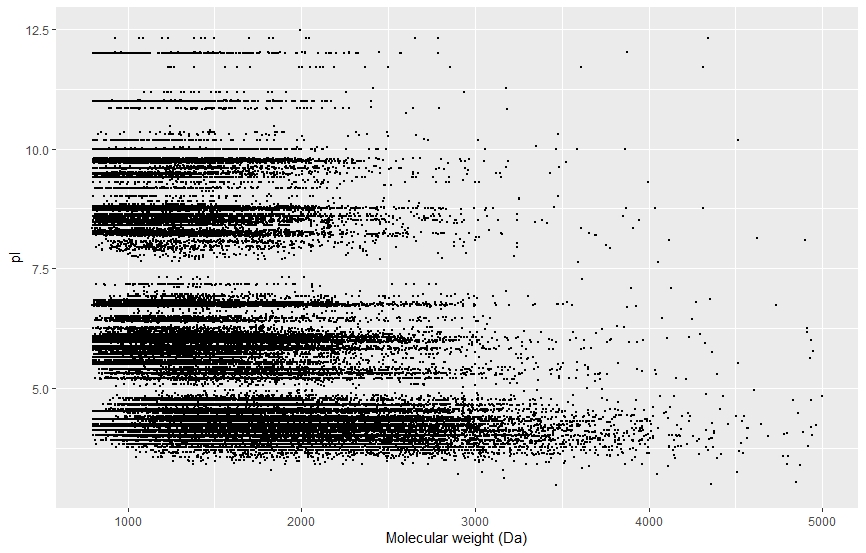** |
| **(f)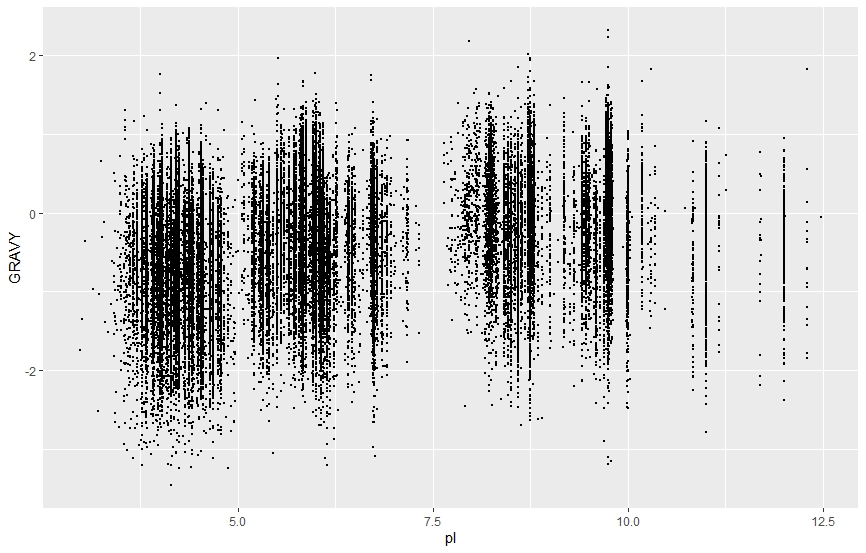** |

**Figure S1.** Relationship between different parameters of identified peptides. (a) Molecular weight (MW) and retention time. (b) Isoelectric point (pI) and retention time. (c) The grand average of hydropathy (GRAVY) value and retention time. (d) GRAVY and MW. (e) pI and MW. (f) GRAVY and pI.

| 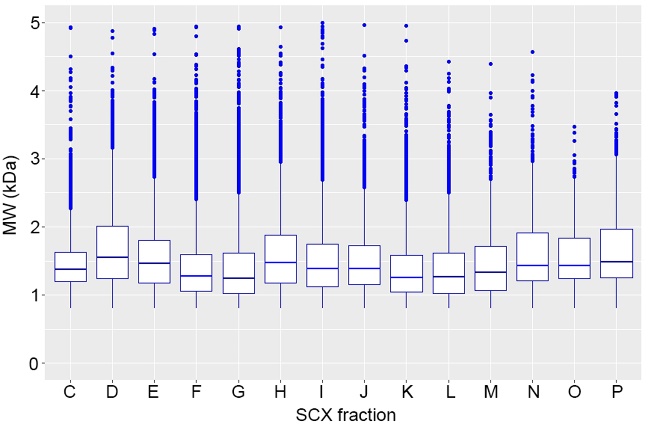 |
| --- |
| (**a**) |
| **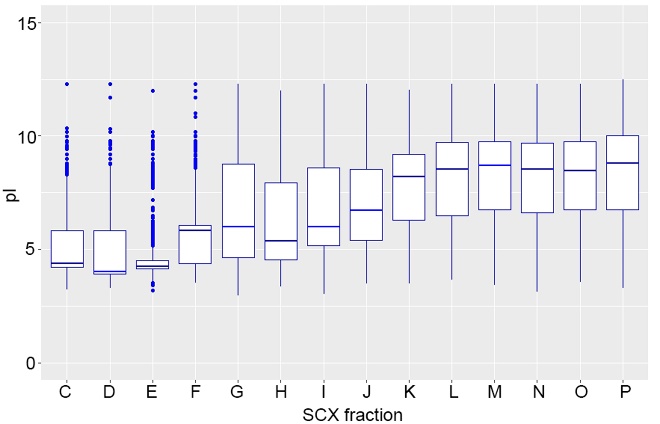** |
| (**b**) |
| **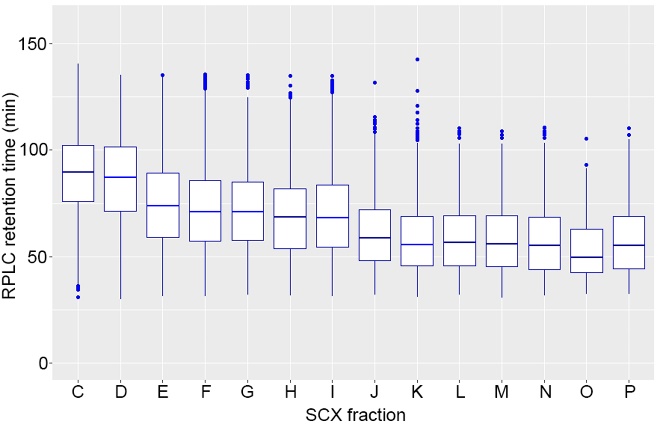** |
| (**c**) |
| **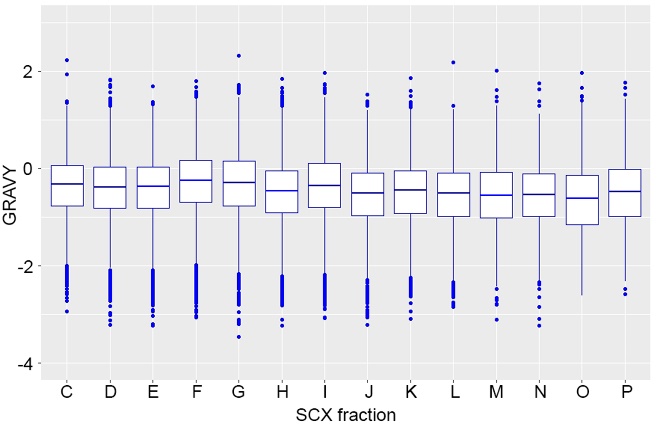** |
| (**d**) |

**Figure S2.** Effect of SCX fractionation on distribution of (a) molecular weight (MW), (b) isoelectric point (pI), (c) RPLC retention time, and (d) the grand average of hydropathy (GRAVY) value of identified peptides.
